# Reference ranges for TSH and thyroid hormones

**DOI:** 10.1186/1756-6614-8-S1-A17

**Published:** 2015-06-22

**Authors:** Krzysztof Lewandowski

**Affiliations:** 1 Department of Endocrinology and Metabolic Diseases, Polish Mother’s Memorial Research Institute, The Medical University of Lodz, Lodz, Poland

## 

Though TSH remains the most commonly used endocrine test in clinical practice, the issue of an appropriate TSH, and to a lesser extent, free T4 and free T3 reference ranges is still under debate. First of all the distribution of TSH reference range is not normal, with median values (also depending on population iodine intake) usually between 1-1.5 mU/L [1-3]. On the other hand, upper TSH reference limit is (assay-dependent) usually around 4.2-4.5 mU/L. There is also an argument that significant number of patients (up to 30%) with TSH above 3.0 mU/L have an occult autoimmune thyroid disease [4]. On the other hand the upper normal TSH reference range in elderly subjects (i.e. 70 plus years) may extend up to around 6.0 mU/L, with relative small changes in free hormones. This phenomenon also applies to elderly patients, where autoimmune thyroid disease had been ruled out [1,2]. Furthermore, there is little evidence of any adverse effects of autoimmune thyroid disease in the elderly in the absence of an overt hypothyroidism. Indeed, Wang et al [5] surprisingly demonstrated decreased frailty in elderly subjects with raised titres of thyroid autoantibodies. It is also well known that in the setting of subclinical hypothyroidism, concentrations of free T4 start to decline below 10 pmol/L only with TSH above 6-7 mU/L in elderly subjects, though a decline in free T4 is more pronounced in the young (particularly in those aged 20-29 years), where free T4 starts to decline for TSH concentrations above 4.5 mU/l [6]. Another issue pertains to the risk of cardiovascular disease in subjects with subclinical hypothyroidism. There are data from post-menopausal women enrolled into the Women Health Initiative study, where an increase in the risk of myocardial infarction was demonstrated only for TSH concentrations above 7.0 mU/L [7].

A separate issue, extensively discussed elsewhere [8], pertains to alterations of TSH and free thyroid hormones during pregnancy. In particular there is decrease in TSH in the first trimester followed by a gradual return to pre-pregnancy values. In contrast, there is an increase in free T4 in the first trimester, while in the third trimester free T4 concentrations are usually at the lower end of the standard (i.e. pre-pregnancy) reference range, or even just below the lower reference limit. Hence, there is an urgent need (postulated in the Endocrine Society Guidelines [8]) to create separate reference ranges for TSH and free thyroid hormones during pregnancy, possible with reference to each trimester. There are also recent data from China [9], where the authors demonstrated that the above-mentioned first trimester-related alterations in TSH and free T4, become only really visible after about 5^th^-6^th^ week of gestation. The authors of this study suggest that standard reference ranges for TSH and free T4 can be applied up to this point of gestation.
